# Supplementary material for: Moving beyond the ‘CAP’ of the Iceberg: Intrinsic connectivity networks in fMRI are continuously engaging and overlapping
Source: Neuroimage. Author manuscript; Available in PMC 2022 May 15. (PMC9107614; doi:10.1016/j.neuroimage.2022.119013)

Supplementary 1. Similarity between subject-level DMN and group-level DMN for Node_Seed1_

| Node_Seed1_ | AllTPs | EPTs | EATs |
| --- | --- | --- | --- |
| ICA | 0.674 ± 0.053 | 0.481 ± 0.073 | 0.356 ± 0.077 |
| FCM | 0.427 ± 0.097 | 0.260 ± 0.166 | 0.289 ± 0.185 |
| ASM | 0.407 ± 0.109 | 0.275 ± 0.088 | 0.254 ± 0.184 |
| wASM | 0.425 ± 0.110 | 0.286 ± 0.090 | 0.283 ± 0.196 |

Supplementary 2. The default mode functional patterns were obtained by performing analyses using the absolute value of Node_Seed1_ time series. The spatial maps are displayed at their peak activation coordinates. AllTPs: all time points, ETPs: the event present time points, EATs: event absent time points (EATs). ICA: independent component analysis, FCM: functional connectivity map, ASM: Activation spatial map, wASMs: weighted ASMs


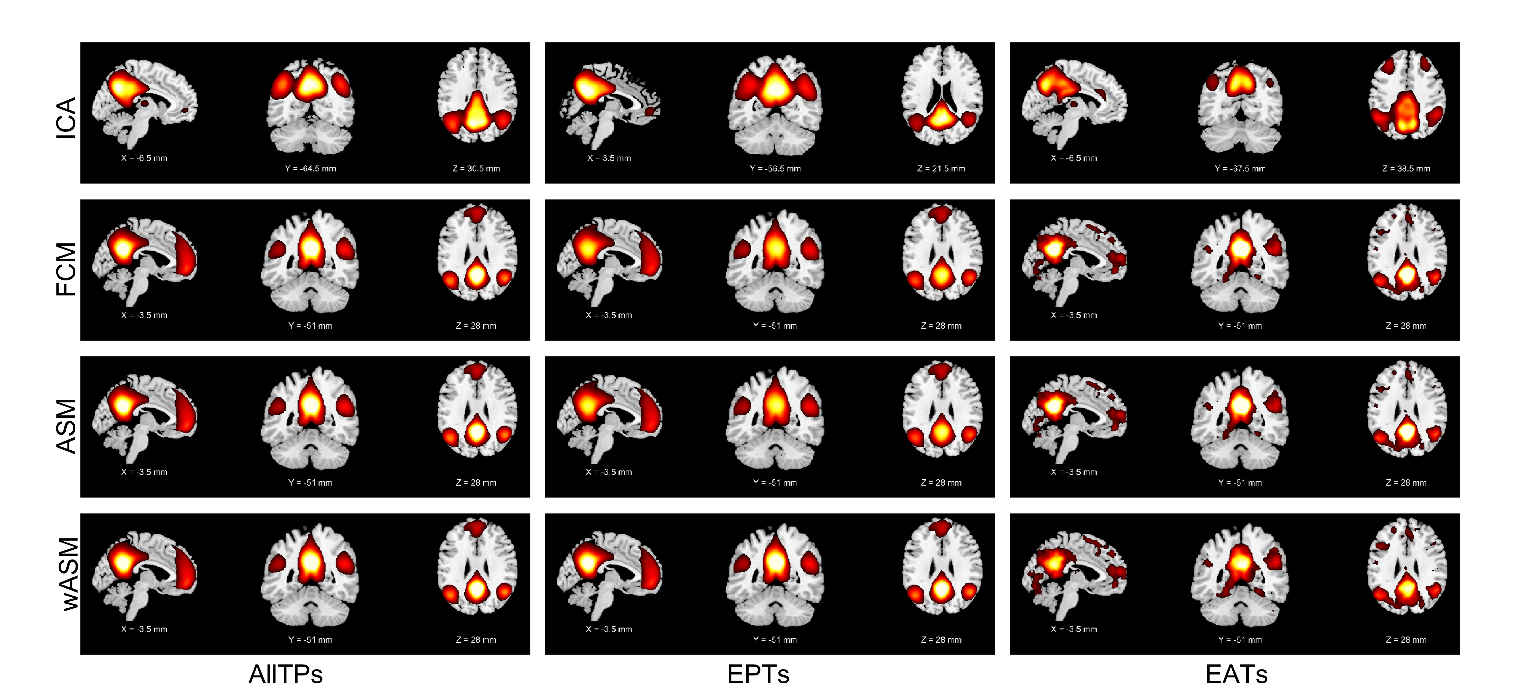


Supplementary 3. The default mode functional patterns were obtained by performing analyses using the amplitude of Node_Seed2_ time series. The spatial maps are displayed at their peak activation coordinates. AllTPs: all time points, ETPs: the event present time points, EATs: event absent time points (EATs). ICA: independent component analysis, FCM: functional connectivity map, ASM: Activation spatial map, wASMs: weighted ASMs


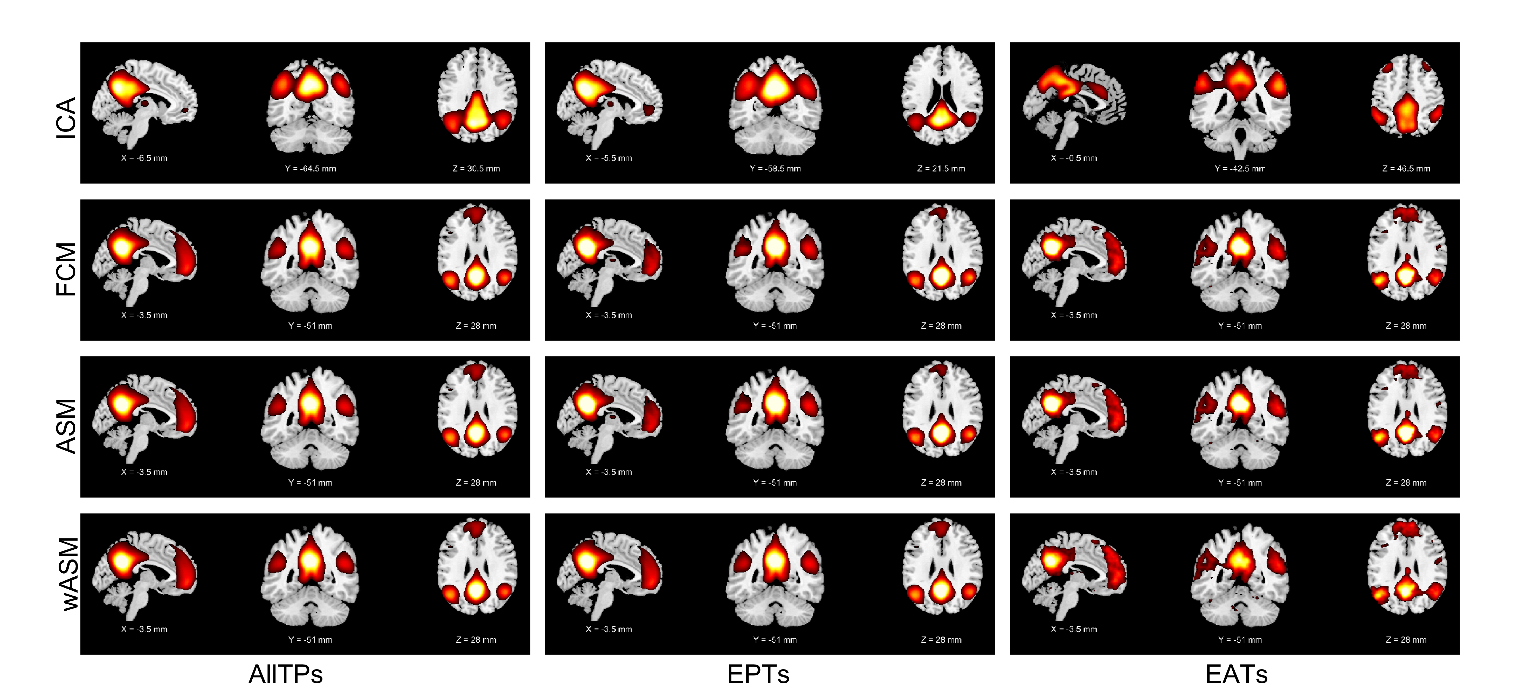


Supplementary 4. The default mode functional patterns were obtained by performing analyses using the amplitude of Node_Meta_ time series. The spatial maps are displayed at their peak activation coordinates. AllTPs: all time points, ETPs: the event present time points, EATs: event absent time points (EATs). ICA: independent component analysis, FCM: functional connectivity map, ASM: Activation spatial map, wASMs: weighted ASMs


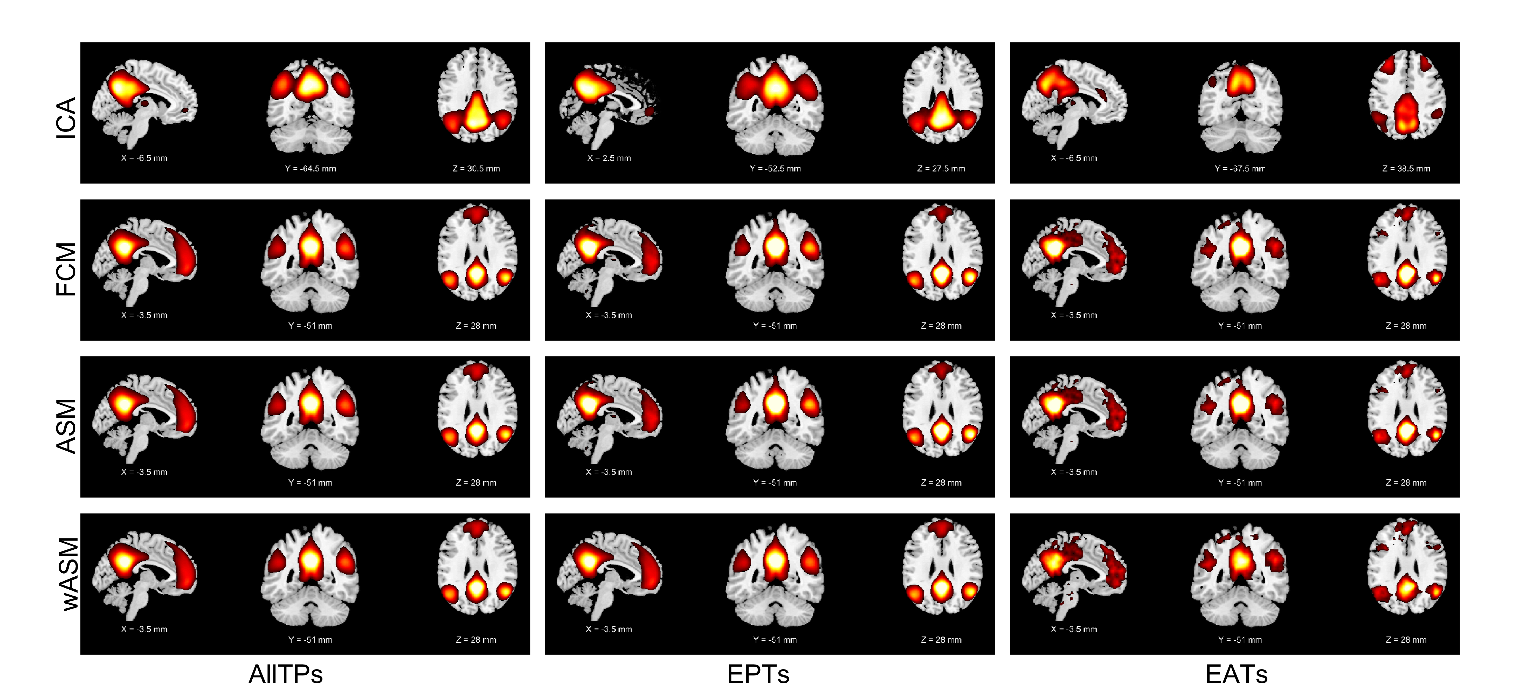


Supplementary 5. The spatial similarity between the default mode was obtained using Node_Seed1_ and Node_Meta_: “subject-level mean ± subject-level standard deviation (group-level)”.

|  | AllTPs | EPTs | EATs |
| --- | --- | --- | --- |
| ICA | 1.000 ± 0.000 (1.000) | 0.842 ± 0.102 (0.994) | 0.496 ± 0.160 (0.944) |
| FCM | 0.909 ± 0.076 (0.971) | 0.689 ± 0.221 (0.968) | 0.460 ± 0.244 (0.961) |
| ASM | 0.863 ± 0.098 (0.975) | 0.744 ± 0.178 (0.982) | 0.399 ± 0.233 (0.962) |
| wASM | 0.914 ± 0.078 (0.977) | 0.807 ± 0.160 (0.983) | 0.468 ± 0.251 (0.965) |

Supplementary 6. The sorted contribution of the visual (A) and somatomotor (B) networks to the BOLD signal over time. Similar to the default mode (Figure 1), the results show smooth changes for the contributions of the visual and somatomotor to time points with no sudden change that can explain changes related to before and after the occurrence of an event.


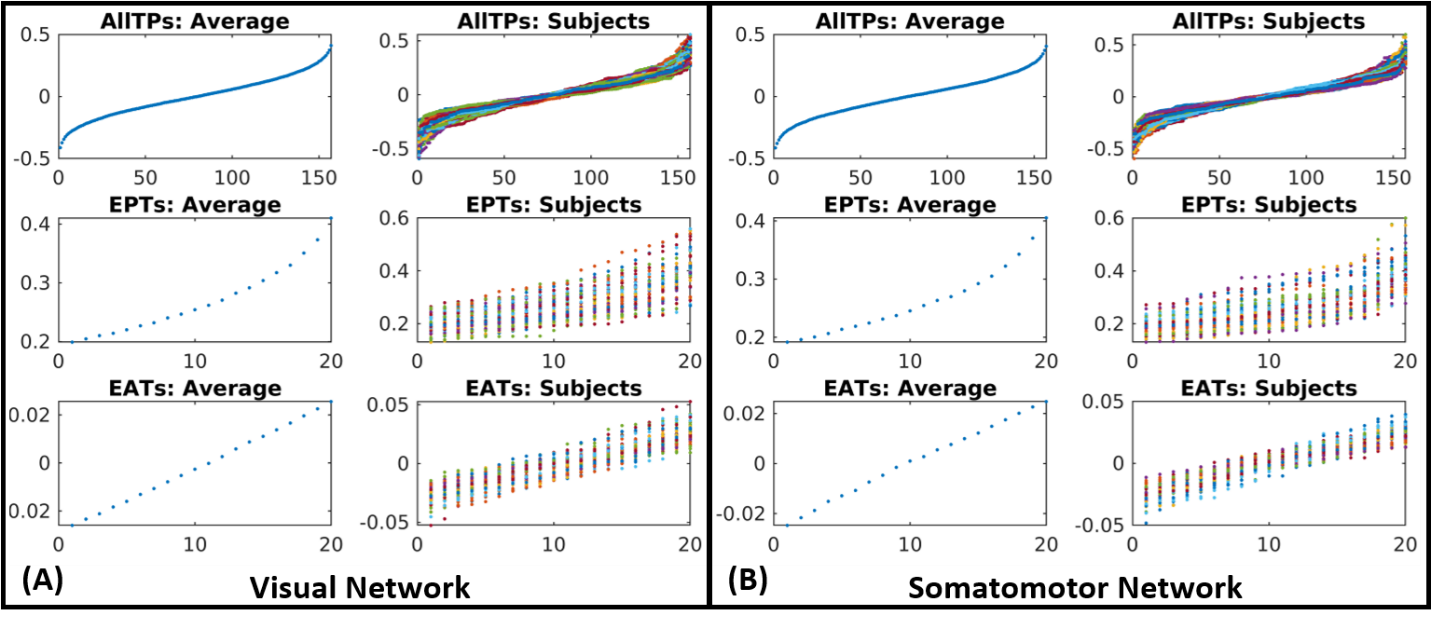


Supplementary 7. Performing analysis for the visual and motor networks using Nodes_Meta_ obtained from Neurosynth. Results show the fidnings are not limited to the default mode and other networks can be obtained from EATs.


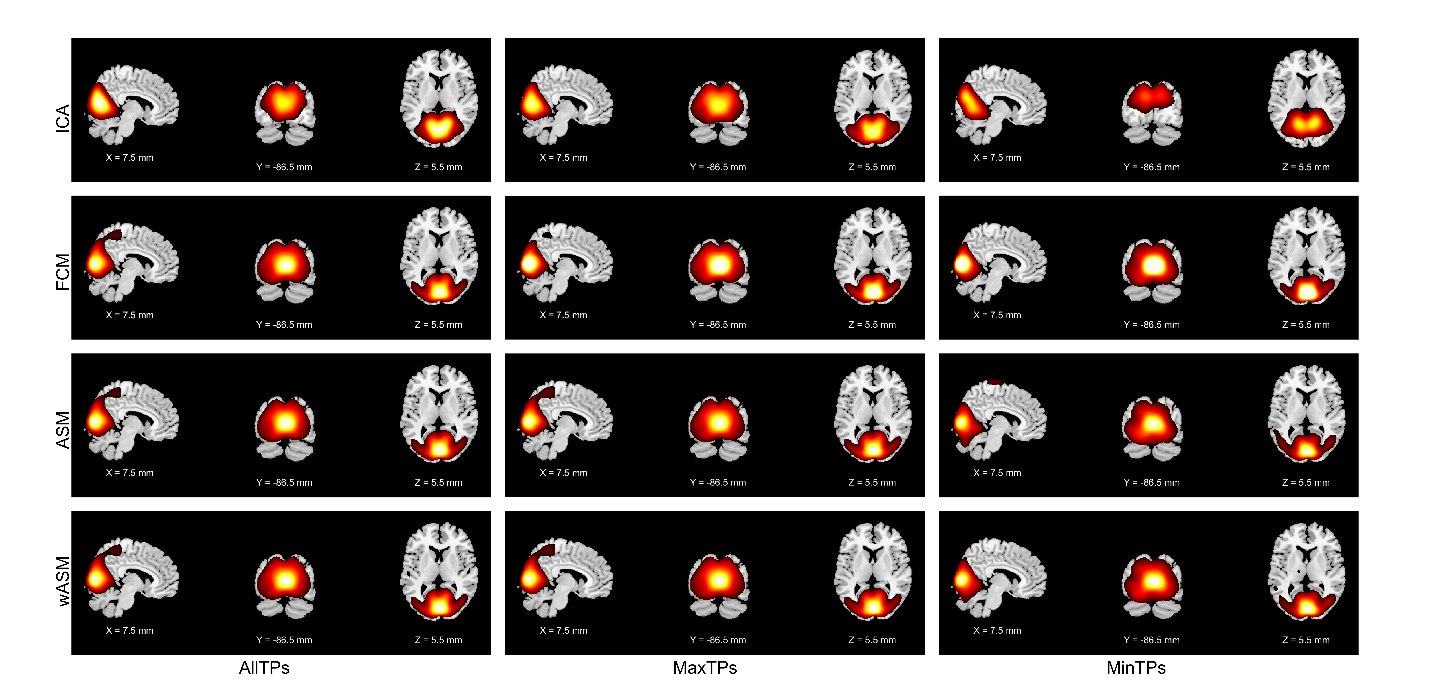


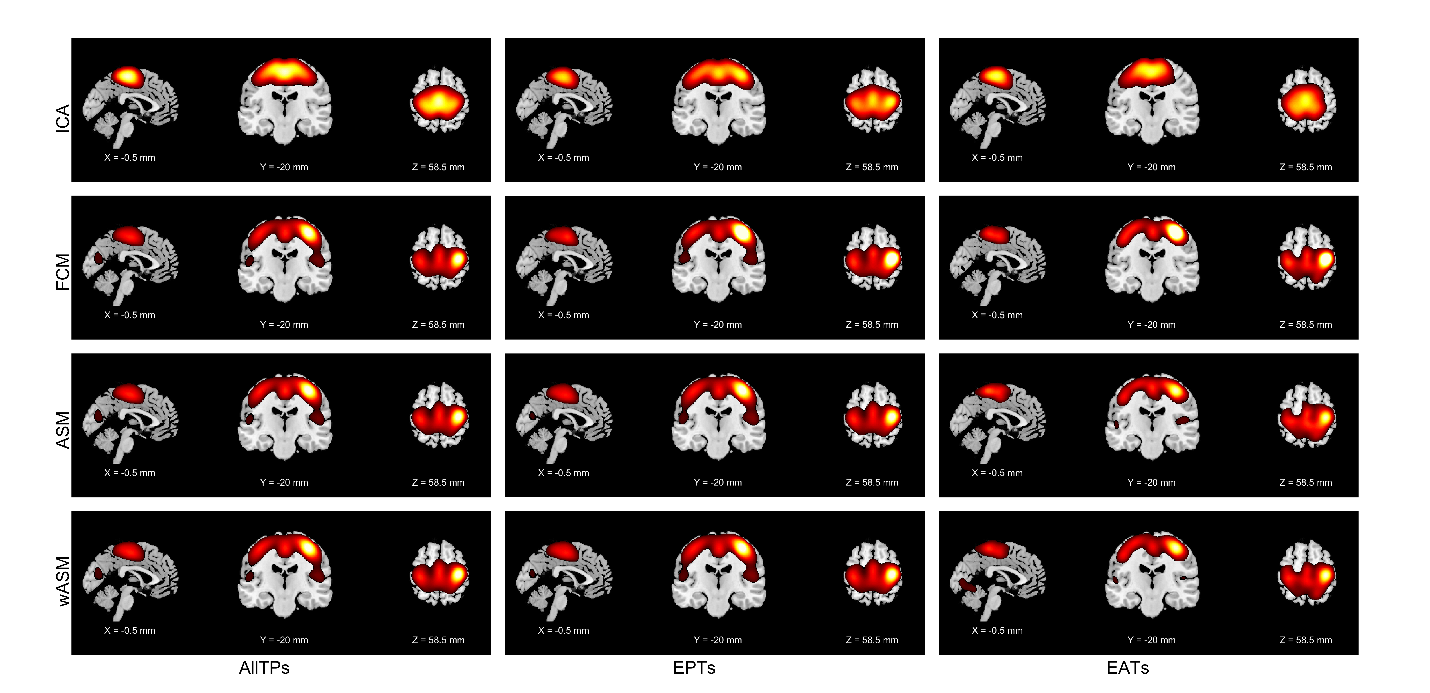


Supplementary 8. The large-scale networks calculate using the absolute value of Node_Seed1_ time courses. The spatial maps are displayed at their peak activation coordinates. AllTPs: all time points, ETPs: the event present time points, EATs: event absent time points (EATs). ICA: independent component analysis, FCM: functional connectivity map, ASM: Activation spatial map, wASMs: weighted ASMs


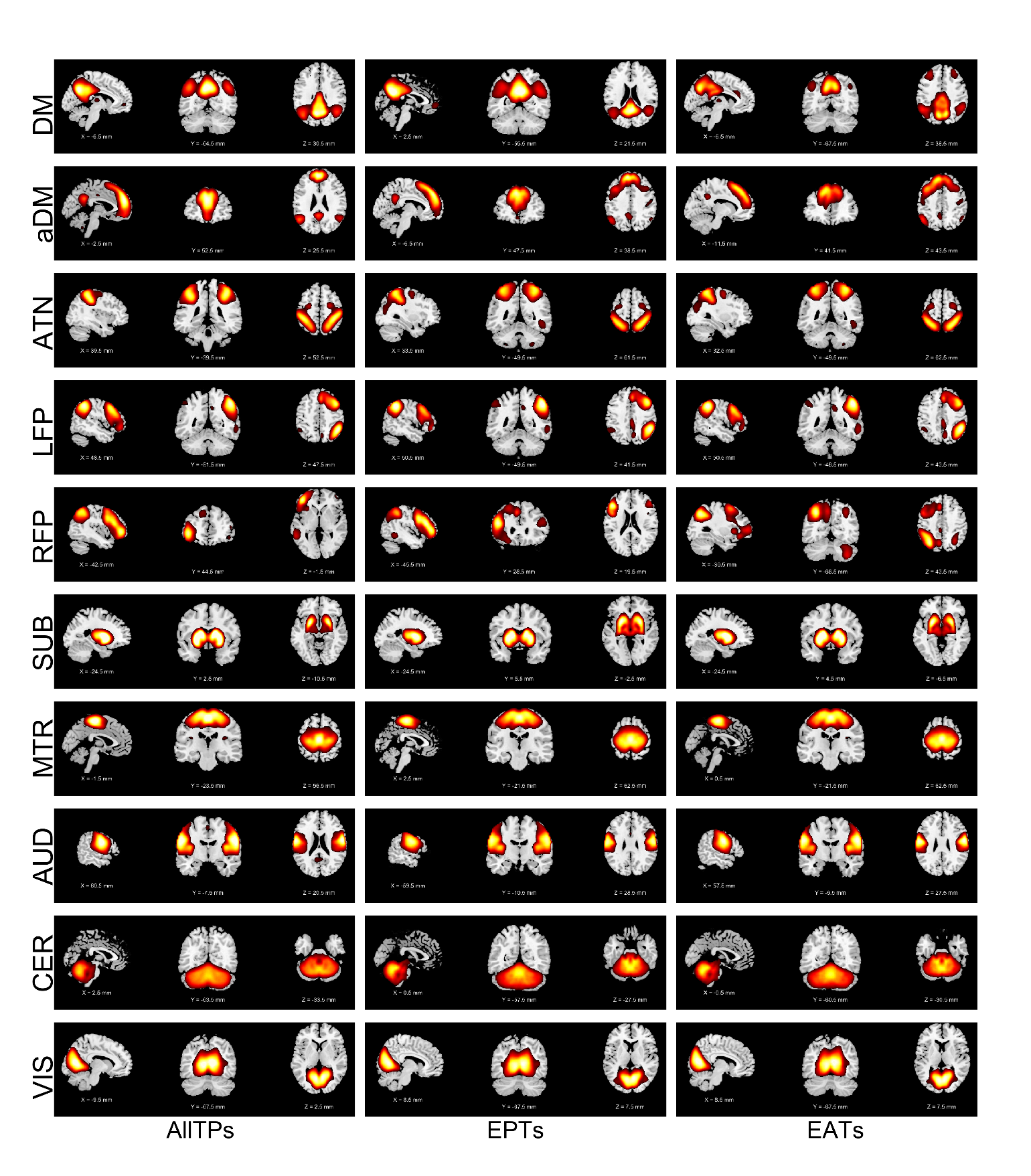


Supplementary 9. The large-scale networks calculate using the absolute value of Node_Seed2_ time courses. The spatial maps are displayed at their peak activation coordinates. AllTPs: all time points, ETPs: the event present time points, EATs: event absent time points (EATs). ICA: independent component analysis, FCM: functional connectivity map, ASM: Activation spatial map, wASMs: weighted ASMs


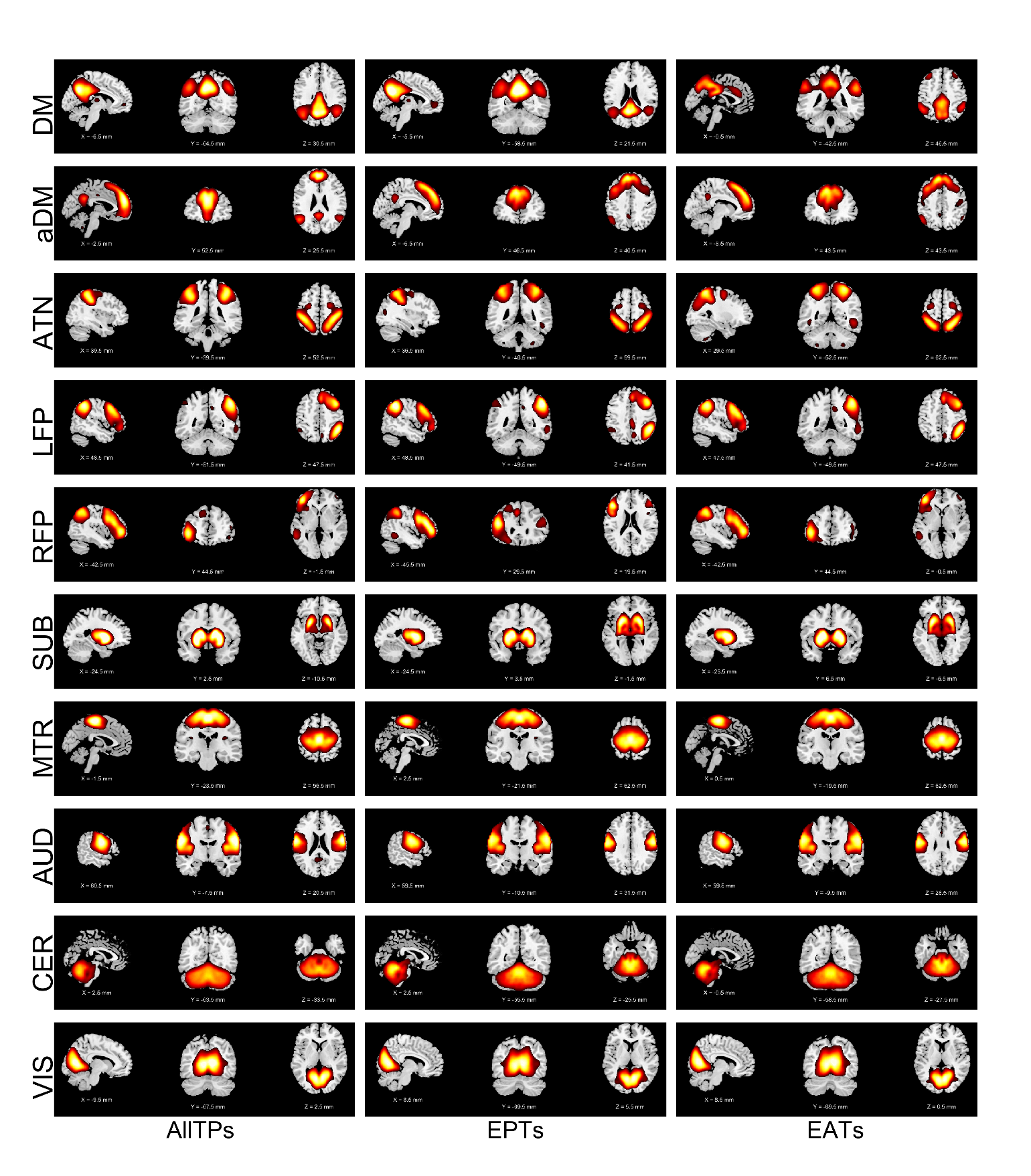


Supplementary 10. The default mode voxel-wise group comparison between typical controls and individuals with schizophrenia, while including age, gender, site, and mean framewise displacement (mFD) as confound regressions and correcting for multiple comparisons. Results show a significant decrease in the thalamus area in EATs, but not for EPTs and AllTPs.


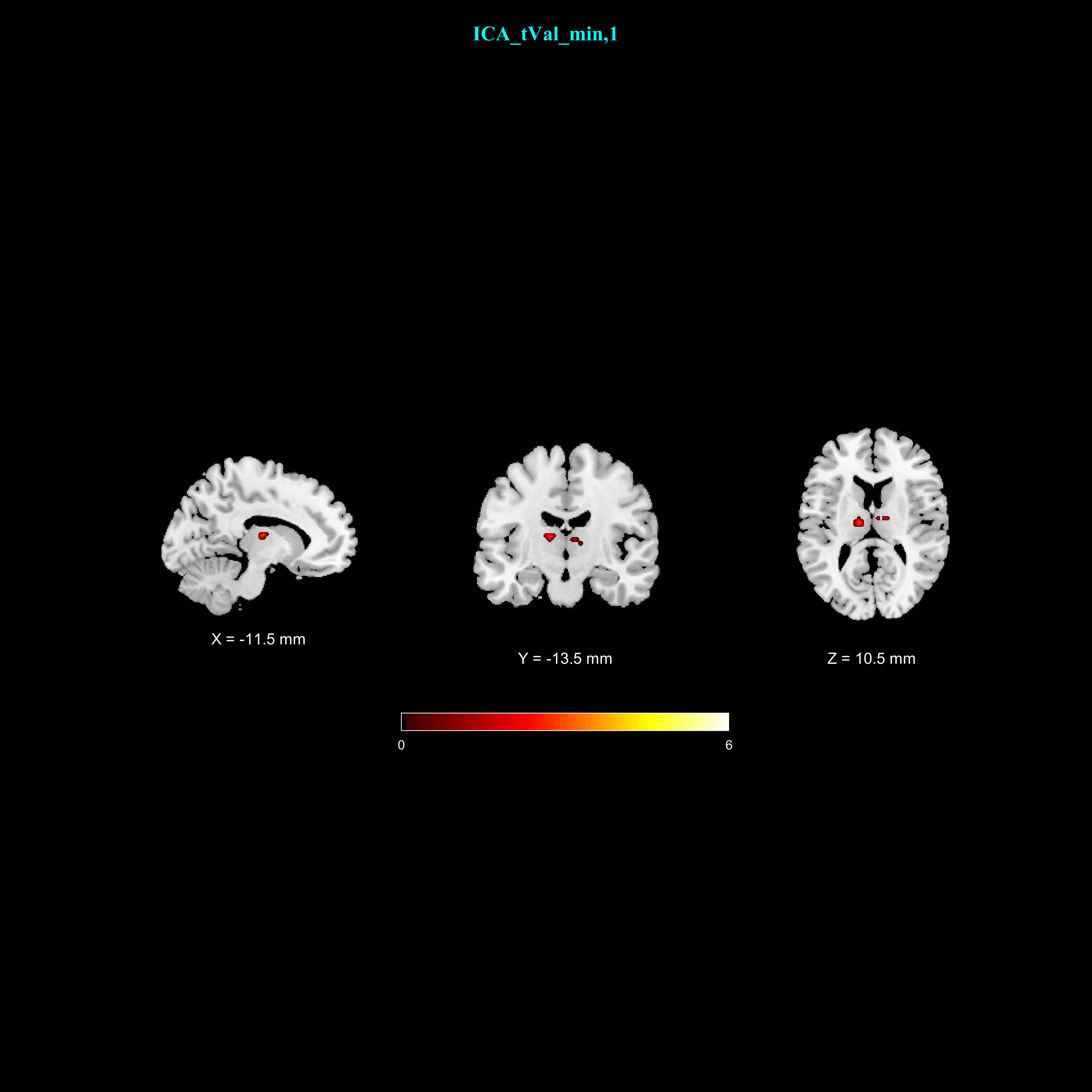

Supplement: 1 [file NIHMS1792866-supplement-1.docx]
